# The Causes of Appendicitis

**Published:** 1905-03

**Authors:** Joseph N. LeConte

**Affiliations:** Atlanta, Ga., Gastro-enterologist to Presbyterian Hospital


ATLANTA

### Journal-Record of Medicine.

Successor to Atlanta Medical and Surgical Journal, Established 1855, and Southern Medical Record, Established 1870.

OWNED BY THE ATLANTA MEDICAL JOURNAL CO.

Published Monthly.

VOL. VI. MARCH, 1905. No. 12.

BERNARD WOLFF, M.D., EDITOR, Nos. 319-20 Prudential.

M. B. HUTCHINS, M.D., BUSINESS MANAGER, 1007-1008 Century Bldg.

J. N. LeCONTE, M.D., ASSOCIATE EDITOR.

### ORIGINAL COMMUNICATIONS.

### THE CAUSES OF APPENDICITIS.*

JOSEPH N. LeCONTE,
ATLANTA, GA.,
Gastro-enterologist to Presbyterian Hospital.

The vermiform appendix, a musculo-membranous tube attached to the posterior and inferior surface of the cecum, and supposed to be a withered relic of the immense ceca of our prehistoric ancestors, has in recent years come into prominence on account of the alarming frequency of the disease which involves the appendix and adjacent structures.

Those forms of animal life which subsist largely on fibrous plant-food have ceca of large dimensions, in some cases overshadowing the stomach in size and importance of digestive function. If we trace the changes in animals further, we find that the carnivora

*Read as a part of a symposium on Appendicitis before Atlanta Society of Medicine, February 16, 1905.

have a cecum rudimentary in size and function; man being omnivorous possesses a cecum of moderate development, and suited to his needs.

Typhlitis, perityphlitis and paratyphlitis, which were more or less fully described in the literature prior to 1880, are now known to originate, in the great majority of cases, in an inflammation of the appendix. The factors which are believed to cause appendicitis are so interwoven with and dependent upon the anatomy and histology of the cecum and appendix that a brief review is necessary.

The appendix varies widely in shape, size and position, being generally about three inches in length, slightly curved and of the size of the classical goose-quill; attached at one end to the cecum, its blind extremity hangs loose in the abdominal cavity, but frequently lies within and behind the cecum. Its mesentery, which is occasionally absent, varies in length, being usually shorter than the appendix and carries lymphatics and nerves, and a branch of the superior mesenteric artery, which in the male is a terminal artery, but in the female has an anastomosis with another artery borne thereto in the appendiculo-ovarian ligament.

The appendix resembles the small intestine in its blood and nerve supply and in the structure of its walls, being usually entirely covered with peritoneum. The lumen of the appendix which is about two-tenths of an inch in diameter is closed more or less perfectly at its junction with the cecum by a semicircular fold of mucus membrane, "the valve of Gerlach."

The causes of appendicitis may be classed as predisposing and exciting, the anatomy of the appendix and its relation to adjacent structures being the predisposing causes, and the exciting causes being largely manifested in mechanical and bacterial agencies.

With regard to age a very large percentage of cases are seen in the second and third decades of life, small children and the aged being peculiarly exempt. As age advances the mucus and submucus layer of the appendix sometimes undergo a process of involution, by means of which the glandular and lymphoid structures are lost and a hyperplasia takes its place, and the lumen is entirely obliterated. So frequently is this found in the aged that it is claimed by some to be a physiological process.

Appendicitis is more prevalent in men than women in the proportion of three to one. This has never been satisfactorily explained, though Bryant states that the appendix is longer and has a larger lumen in the male than the female and would more easily permit the entrance and retention of intestinal contents; moreover the additional blood supply in the female is probably counterbalanced by the likelihood of inflammation extending from the uterus or adnexa.

As to occupation, those who lift heavy weights or are exposed to traumatism seem to be more subject to attack. The extension of certain disease processes into the appendix such as is seen in dysentery, tuberculosis, typhoid fever, syphilis or actinomycosis occasionally cause appendicitis, though they rarely present the typical symptoms generally seen in that condition. Excessive use of alcoholics, exposure to cold, influenza, rheumatic diathesis and many other influences are invoked as causes.

Foreign bodies were formerly thought to be a frequent cause of the disease, but statistics show that hardly two per cent. of autopsies or operations in this condition reveal foreign bodies. Fecal concretions hard or soft are frequently present and certainly act as an exciting cause in some cases, though they are often found in the normal appendix. A fecal concretion may pass into the appendix fully formed, or may be built upon a fruit seed or particle of mucus. These may cause inflammation in either of two ways, first on account of pressure, ulceration may take place at the point of contact, but more often they cause retention and stagnation of the appendical contents, and infection follows, operation often revealing the perforation in the appendical wall distal to the concretion.

It is a well-recognized fact that when pressure is exerted upon any tissue it becomes subject to the onslaught of pathogenic organisms which otherwise would continue harmless on its surface. We can readily see that the same condition could be brought about by a catarrh of the colon or cecum from any cause such as fecal stasis or chemical or mechanical irritants, an extension of which catarrh into the appendix could cause occlusion of lumen, stagnation of contents and infection.

Distention of the cecum of ileum with gas or fecal matter may act as an exciting cause. Where unusual growth or overdistention of the lower ileum or cecum occurs, the enlargement takes place at the expense of the narrow mesentery of the appendix and causes distortion, constriction and interference of blood supply.

Rubin, of Chicago, has recently set forth the voluntary retention of intestinal gases as the chief predisposing cause of appendicitis, and fortifies his position by experiments on ten cadavers, the ileum coecum and part of the colon of which were first cleansed, then ligated after the introduction of shot, peas and beans of various sizes. Manipulation was then practiced to imitate peristalsis as well as possible, but none of the foreign bodies entered the appendix in any of the subjects; after inflation with air, (which by the way easily ballooned the appendix) manipulation was again practiced, and resulted in the entrance of numerous shot in every appendix but one, and in some cases peas entered also.

Rubin suggests as an explanation of his theory "the accumulation of gases below the ileocecal valve, and their voluntary retention, the ensuing distention of the cecum, and dilatation in various degrees of the ceca appendicular orifice, the entrance into the appendix of larger fecal masses than are readily expelled, the interference with the vascular supply and resulting erosion of the mucosa with subsequent infection of the appendix." This, he thinks, would explain its extraordinary prevalence between the ages of ten and thirty when school, social and business life claim so much of time, and surroundings render necessary the voluntary retention of any flatus present: explaining also the rarity of the disease among small children, the semi-civilized races and the insane, among whom excesses in diet are frequent, and social requirements are less heeded. In view of the fact that so many cases reveal no concretions we might look to the constriction of the lumen and interference of blood-supply caused by the distension of the coecum as a cause.

Sir William McEwen, of Glasgow, has recently added a valuable contribution to the literature on this subject, which sets forth the functions of the cecum and appendix, and shows that they have a much more important part in the final stages of digestion than has heretofore been accorded them by physiologists. He deplores the growing tendency among some surgeons to remove the normal appendix when operating for other conditions, and brings forth much to prove that it is not the useless structure hitherto believed.

In a series of six cases operated on by him where the entire cecum had been removed all of the patients suffered thereafter from frequent and troublesome attacks of diarrhea when general diet was taken, and ultimately appeared thin and poorly nourished. It is well known that when an intestinal fistula exists above the cecum, allowing the contents to escape freely, a condition of inanition rapidly supervenes, while a colonic fistula permits the maintenance of a very fair nutrition.

In a series of cases where defects in the abdominal wall and the wall of the cecum laid bare the interior, careful study revealed that there is an intermittent secretion poured out from the glandular structures of the coecum and appendix which appears to be reflexly stimulated by the presence of food above the ileocoecal valve, and that the valve is also controlled reflexly, only permitting a small amount of intestinal contents to pass into coecum at a time. This probably resembles the acid reflex of the pylorus described by Pawlow.

The muscular structure of the appendix is analogus to that of the coecum, the circular fibres being directly continuous with those of the coecum and the longitudinal bands of the cecum continue on the appendix and are lost in its longitudinal fibres, but the vascular and nerve supply correspond to that of the small intestine. McEwen has found that the peristaltic movements of the cecum originate in the appendix and are continued into the cecum, and there is good reason to believe that this action of the appendix is controlled by a reflex impulse from the small intestines through the superior mesenteric plexus of nerves.

The glands of the Lieberkuhn are larger and more numerous in the cecum and appendix than any part of the intestinal tract; so closely are they placed in the mucus membrane that they leave little room for absorbing surface, and prove conclusively that the cecum and appendix were intended for secreting much more than for absorbing.

The secretion of these Lieberkuhn's glands has been proven in recent years to have an important function in aiding the pancreatic juice to digest foods, especially proteids: coagulated egg white, which is not attacked by pancreatic juice after ten hours in a thermostat, is dissolved in six minutes by the addition of succus entericus. The important action of micro-organisms in the cecum and colon of disentegrating resistant portions of food such as cellulose and converting them into soluble glucose, etc., is generatly recognized, and the appendix, which always contains these micro-organisms, maintains them in propor culture and periodically ejects a sufficient amount to carry on this disintegration in the coecum and colon.

Those things which interfere with the action of the ileocecal valve, the secretion of the cecum and appendix, the peristaltic action of the appendix, and the control of the development of the micro-organisms in the appendix, tend to produce cecal indigestion, stasis and appendicitis.

One often hears among the laity the rumor that the removal of the appendix carries in its wake ill health and early demise. A series of careful statistics relating to the condition of health of a large number of individuals after removal of the appendix; their tendency to diarrhea, constipation, poor nutrition, and the necessity of a specially selected diet to prevent these conditions, would throw much light on the function of appendix and the causes of its frequent inflammatory involvement.

In conclusion, though the causes of appendicitis are varied, the majority are intimately associated with the digestive process and the factors controlling it; no less an authority than Deaver asserts that, "appropriate inquiry will elicit a history of such disturbances in almost all cases."

